# Feasibility of aspirin and/or vitamin D3 for men with prostate cancer on active surveillance with Prolaris® testing

**DOI:** 10.1002/bco2.169

**Published:** 2022-06-11

**Authors:** Eoin Dinneen, Gregory L. Shaw, Roseann Kealy, Panos Alexandris, Kier Finnegan, Kimberley Chu, Nadia Haidar, Sara Santos‐Vidal, Sakunthala Kudahetti, Caroline M. Moore, Alistair D. R. Grey, Daniel M. Berney, Anju Sahdev, Paul J. Cathcart, R. Timothy D. Oliver, Prabhakar Rajan, Jack Cuzick, for the PROVENT study group, Jack Cuzick, Sanjeev Madaan, Jhumur Pati, Abdul M. Chowdhury, Brian R. P. Birch, Timothy J. Dudderidge, Caroline M. Moore, Alistair D. R. Grey, Gregory L. Shaw, Kieran P. Jefferson, Howard G. Kynaston, Prabhakar Rajan, James S. A. Green, Paul J. Cathcart, Daniel M. Berney, Thomas Powles, R. Timothy D. Oliver, Anju Sahdev, Roseann Kealy, Victoria Kemp, Panos Alexandris, Kier Finnegan, Kimberly Chu

**Affiliations:** ^1^ Division of Surgery and Interventional Science University College London London UK; ^2^ Department of Urology, University College Hospital at Westmoreland Street University College Hospital London NHS Foundation Trust London UK; ^3^ Centre for Prevention, Detection and Diagnosis, Wolfson Institute of Population Health, Cancer Research UK Barts Centre Queen Mary University of London London UK; ^4^ Department of Urology, The Royal London Hospital Barts Health NHS Trust London UK; ^5^ Centre for Cancer Biomarkers and Biotherapeutics, Barts Cancer Institute, Cancer Research UK Barts Centre Queen Mary University of London London UK; ^6^ Department of Cellular Pathology, The Royal London Hospital Barts Health NHS Trust London UK; ^7^ Department of Radiology, St Bartholomew's Hospital Barts Health NHS Trust London UK; ^8^ Department of Urology, Guy's Hospital Guy's and St Thomas' NHS Foundation Trust London UK; ^9^ Centre for Cancer Cell and Molecular Biology, Barts Cancer Institute, Cancer Research UK Barts Centre Queen Mary University of London London UK; ^10^ Present address: Cancer Prevention Trial Unit, School of Cancer & Pharmaceutical Sciences King's College London London UK.; ^11^ Department of Urology, Darent Valley Hospital Darent and Gravesham NHS Trust Dartford UK; ^12^ Department of Urology, Homerton University Hospital Homerton University Hospital NHS Foundation Trust London UK; ^13^ Department of Urology, Southampton General Hospital University Hospitals Southampton NHS Foundation Trust Southampton UK; ^14^ Department of Urology, University Hospital Coventry University Hospitals Coventry and Warwickshire NHS Trust Coventry UK; ^15^ Department of Urology University Hospital of Wales Cardiff UK

**Keywords:** active surveillance, adjunctive therapy, aspirin, biomarkers, prostate cancer, vitamin D

## Abstract

**Objectives:**

To test the feasibility of a randomised controlled trial (RCT) of aspirin and/or vitamin D3 in active surveillance (AS) low/favourable intermediate risk prostate cancer (PCa) patients with Prolaris® testing.

**Patients and Methods:**

Newly‐diagnosed low/favourable intermediate risk PCa patients (PSA ≤ 15 ng/ml, International Society of Urological Pathology (ISUP) Grade Group ≤2, maximum biopsy core length <10 mm, clinical stage ≤cT2c) were recruited into a multi‐centre randomised, double‐blind, placebo‐controlled study (ISRCTN91422391, NCT03103152). Participants were randomised to oral low dose (100 mg), standard dose (300 mg) aspirin or placebo and/or vitamin D3 (4000 IU) versus placebo in a 3 × 2 factorial RCT design with biopsy tissue Prolaris® testing. The primary endpoint was trial acceptance/entry rates. Secondary endpoints included feasibility of Prolaris® testing, 12‐month disease re‐assessment (imaging/biochemical/histological), and 12‐month treatment adherence/safety. Disease progression was defined as any of the following (i) 50% increase in baseline PSA, (ii) new Prostate Imaging‐Reporting and Data System (PI‐RADS) 4/5 lesion(s) on multi‐parametric MRI where no previous lesion, (iii) 33% volume increase in lesion size, or radiological upstaging to ≥T3, (iv) ISUP Grade Group upgrade or (v) 50% increase in maximum cancer core length.

**Results:**

Of 130 eligible patients, 104 (80%) accepted recruitment from seven sites over 12 months, of which 94 patients represented the per protocol population receiving treatment. Prolaris® testing was performed on 76/94 (81%) diagnostic biopsies. Twelve‐month disease progression rate was 43.3%. Assessable 12‐month treatment adherence in non‐progressing patients to aspirin and vitamin D across all treatment arms was 91%. Two drug‐attributable serious adverse events in 1 patient allocated to aspirin were identified. The study was not designed to determine differences between treatment arms.

**Conclusion:**

Recruitment of AS PCa patients into a multi‐centre multi‐arm placebo‐controlled RCT of minimally‐toxic adjunctive oral drug treatments with molecular biomarker profiling is acceptable and safe. A larger phase III study is needed to determine optimal agents, intervention efficacy, and outcome‐associated biomarkers.

## 1 INTRODUCTION

Prostate cancer (PCa) is the most common male‐specific cancer and its worldwide incidence is rising^1^ largely due to increased prostate specific antigen (PSA) and multi‐parametric magnetic resonance imaging (mpMRI) utilisation in an ageing population. For many patients with localised low and favourable intermediate risk PCa, radical treatments (RT) (prostatectomy or radiotherapy) will cause treatment‐related toxicity without a survival benefit.^2^ Over 40% of all patients with low risk PCa are managed by active surveillance (AS),^3^, ^4^ which aims to avoid or defer RT guided by serial PSA monitoring, sequential mpMRI, and prostate biopsy.^5^ However, despite modern AS strategies, up to 30%–50% of AS patients opt for RT within 5 years, in many cases due to patient anxiety and not overt disease progression.^6^

Molecular (e.g., PCA3, TMPRSS2:ERG or Prolaris®)^7^, ^8^ and imaging (e.g., mpMRI)^9^ biomarkers may help to risk stratify patients on AS to identify those at higher disease progression risk. Minimally‐toxic adjunctive oral drug treatments given after diagnosis to reduce disease progression could increase long‐term patient AS adherence, thereby diminishing RT‐related toxicity at a population level. In a chemoprevention setting, dietary supplements such as selenium and vitamin E,^10^ and beta‐carotene,^11^ have failed to reduce PCa incidence, and 5‐alpha reductase inhibitors (5ARI)^12^, ^13^ do not reduce development of high‐risk disease. For PCa patients on AS, the 5ARI dutasteride reduces mpMRI‐determined tumour volume for low and intermediate risk disease,^14^ and may decrease low‐grade disease progression risk.^15^

Aspirin, acetylsalicylic acid, is a common, well‐tolerated, non‐steroidal anti‐inflammatory drug (NSAID) with a known side‐effect profile that has been associated with a reduction in PCa incidence and death rates in clinical prevention trials and cohort studies.^16^ However, no direct comparisons of low dose (100 mg) versus standard dose (300 mg) have been undertaken and there is uncertainty whether the low dose anti‐platelet actions of aspirin have sufficient anti‐cancer effects. Vitamin D is a fat‐soluble secosteroid that is linked to calcium homeostasis and bone metabolism but may also have antiproliferative properties.^17^ Higher vitamin D levels have been associated with lower cancer risk,^18^ and higher frequency of sun exposure (which increases activated vitamin D levels) has also been associated with lower PCa risk.^19^ A study of low risk PCa patients on AS treated with 1 year of oral vitamin D supplementation identified a reduction in the number of positive cores at re‐biopsy.^20^

Here, we report outcomes of a feasibility study of a multi‐centre randomised, double‐blind, placebo‐controlled trial investigating oral aspirin and/or vitamin D3 low and favourable intermediate risk PCa patients on AS with tissue biomarker molecular profiling. We wished to explore the feasibility and patient acceptability of recruitment and randomisation, assess compliance and toxicity and determine optimal disease re‐assessment parameters for future definitive studies of these and other minimally‐toxic ‘adjunctive’ treatments.

## 2 PATIENTS AND METHODS

### 2.1 Patients, randomisation and intervention

Patients aged >16 years with newly‐diagnosed low or favourable intermediate risk (PSA ≤ 15 ng/ml, International Society of Urological Pathology (ISUP) Grade Group ≤2, maximum biopsy core length <10 mm, clinical stage ≤cT2c) (Table S1) were enrolled at seven UK sites. Eligible participants were screened at the time of diagnosis, invited to join after choosing AS and recruited after providing written, informed consent. Pre‐biopsy mpMRI was undertaken, and histopathological assessment was performed on tissue samples obtained by transrectal or transperineal prostatic biopsy as per local clinical practice. Patients were not subjected to confirmatory biopsy prior to commencing AS. Post‐randomisation clinical assessments were undertaken as per the study protocol.

**FIGURE 1:**
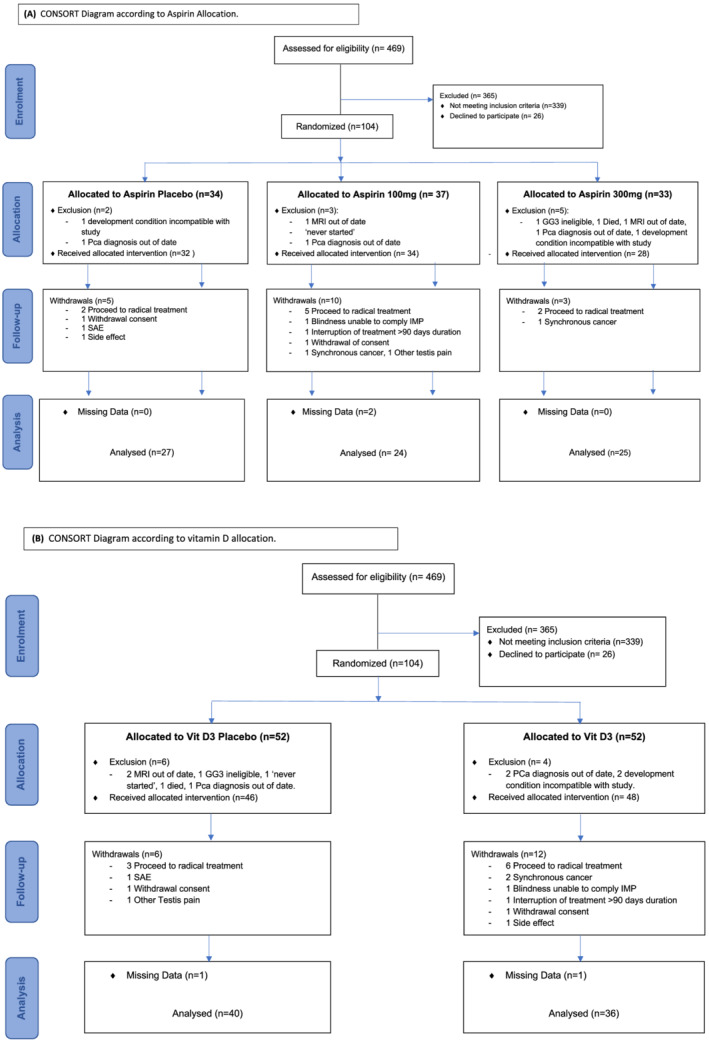
CONSORT diagrams according to aspirin and vitamin D allocation separately

Patients were randomly allocated using a 3 × 2 factorial design via a web‐based randomisation service (hosted by the Barts Clinical Trials Unit) to daily oral low dose (100 mg) or standard dose (300 mg) aspirin versus placebo and/or oral vitamin D3 (4000 IU administered by 8 drops of fluid) versus placebo (see CONSORT Figure 1) with equal numbers in each group. A factorial trial design was employed to (a) evaluate trial design feasibility for future larger studies and (b) for the simultaneous assessment of two treatments in a single study.

Treatment allocation was blinded to participants, clinicians, and the local trial team. Trial prescriptions with concealed labelling were issued at the randomisation visit and every 6 months by site pharmacies. Treatment adherence was assessed as a proportion of dispensed medication that was consumed amongst participants who returned their medication packages for an independent pharmacist review at 6 and 12 months. Patients were followed up for a total of 18 months. PSA testing was performed every 3 months, and repeat mpMRI and re‐biopsy was recommended at 12 months. Adverse events (AEs) and serious AEs (SAE) were recorded prospectively by local site teams and the central trial team was informed. The trial protocol (https://www.isrctn.com/ISRCTN91422391) was approved by the trial sponsor (Queen Mary University of London), Integrated Research Application System (IRAS) (ID 145427), National Research and Ethics Service (REC reference 14/LO/2033) and implemented in accordance with Good Clinical Practice guidelines and the Declaration of Helsinki.

### 2.2 Biomarker assessment

Prolaris® cell cycle profile (CCP) testing was undertaken on surplus diagnostic tissue at baseline and on repeat 12‐month biopsy samples. CCP scores were generated as previously described^21^ by the Myriad Genetics (Salt Lake City, UT, USA) Clinical Laboratory Improvement Amendments (CLIA)‐certified laboratory. Combined cell cycle clinical risk (CCR) scores were calculated as described^7^ by combining the CCP score with the University of California San Francisco (UCSF) Cancer of the Prostate Risk Assessment (CAPRA) score.^22^

### 2.3 Statistical analysis and outcome measurements

A 100 patient sample size was chosen for feasibility with a total recruitment period of 12 months. This was based on an assumption of a 33.3% true recruitment rate from 300 approached eligible individuals, which would achieve 89% power to show that the true recruitment rate is above 25% (two‐sided binomial proportion test with a 0.05 significance level). The principal trial recruitment site diagnoses approximately 750 men with PCa each year, of which around 25% are managed by active surveillance. Six further recruitment sites were included based on geographical diversity. Recruitment data from this feasibility study will inform the number of centres required for a larger trial.

The primary endpoint was trial acceptance and entry rates over a 12‐month period. Secondary endpoints included feasibility of diagnostic tissue biomarker molecular profiling and feasibility of disease re‐assessment at 12 months. Disease progression was defined as any of the following: (i) *biochemical*: 50% increase in serum PSA from baseline; (ii) *radiological*: development of a Prostate Imaging‐Reporting and Data System (PI‐RADS) 4/5 lesion^23^ on mpMRI, where no lesion was identified before, 33% volume increase in the size of the lesion, or radiological upstaging to T3 or above based on local site reports; or (iii) *histological*: upgrade of ISUP Grade Group or a 50% increase in maximum cancer core length based on local site reports. Numbers of patient exclusions, withdrawals, and missing data on disease re‐assessment at 12 months were collected. Further secondary endpoints were tabulation of all SAEs, measurement of serum calcium to compare the effect of vitamin D with placebo, and treatment medication compliance, which was calculated for each participant by study pharmacists blinded to treatment arm allocation at 6 and 12 months.

## 3 RESULTS

**FIGURE 2:**
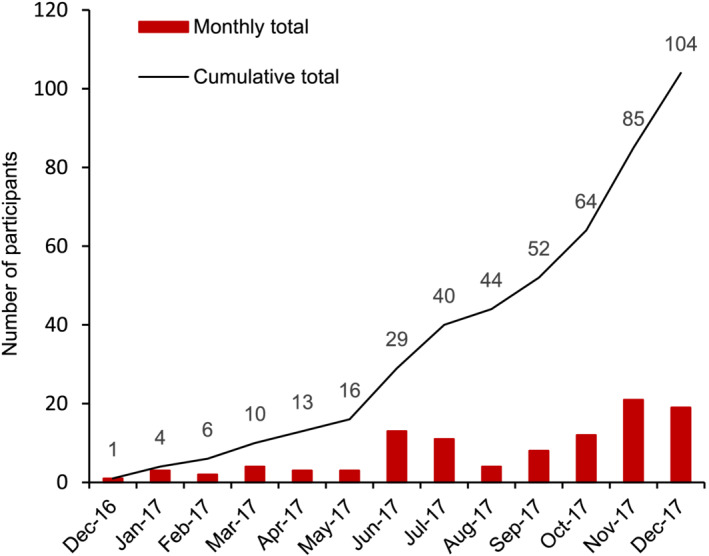
Recruitment of participants by month with cumulative total and monthly increase

Of the 469 men assessed for eligibility at seven sites between December 2016 and December 2017, 130 met the inclusion criteria. The 104 men (80%) accepted recruitment into the study and were randomised to aspirin and/or vitamin D (CONSORT Figure 1A,B). Monthly and cumulative total patient recruitment numbers are shown in Figure 2. Patient baseline demographics are provided in Table 1. Prior to receiving treatment, 10 men were identified as ineligible based on the protocol and were excluded from the study. Reasons are shown in CONSORT Figure 1A,B and are listed in Table S4, creating a per protocol population of 94 men. Prolaris® testing was performed in 76/94 (81%) of baseline diagnostic biopsies where biopsy material was available.

**TABLE 1:** Baseline patient characteristics of 104 participants before exclusions

Variable	*n* (%)	Median (IQR)
Age (years)		61 (55, 66)
<50	9 (8.6)	
50–60	37 (35.6)	
61–70	44 (42.3)	
>70	14 (13.5)	
PSA (ng/ml)		7.2 (5.3, 9.7)
<4	11 (10.6)	
4–10	69 (66.3)	
>10	24 (23.1)	
EAU risk group		
Low	53 (51)	
Intermediate	51 (49)	
High	0	
Biopsy approach		
Transrectal	58 (56)	
Transperineal	46 (45)	
ISUP Grade Group (Gleason Sum Score)		
1 (3 + 3)	82 (78.8)	
2 (3 + 4)	21 (20.6)	
3 (4 + 3)	1 (1)^a^	
CCP score^b^		−0.633 (−1.0, −0.662)
CCR score^b^		0.474 (0.137, 0.45)

Abbreviations: EAU, European Association of Urology; ISUP, International Society of Urological Pathology; PSA, prostate‐specific antigen.

^a^ Patient excluded as ineligible.

^b^ Clinical Progression (CCP) and Clinical Cancer Risk (CCR) scores calculated on 76 of 104 men.

Of all participants, 68.1% (64/94) returned their medication packages for treatment compliance assessment by local trial pharmacy teams at 6 months and 64.5% (49/76) at 12 months (Table S3). Amongst those who returned their medications, treatment adherence was high at both 6 and 12 months. The proportion of aspirin tablets that were consumed across all arms was 91.7% and 91% at 6 and 12 months, respectively. The proportion of vitamin D liquid dispensed that was consumed across all arms was 89% and 91% at 6 and 12 months, respectively (Table S3).

Of eligible patients, 9 men withdrew from the study to have RT before 12 months without evidence of disease progression according to PROVENT trial definitions, and did not undergo 12‐month reassessment. A further nine men withdrew from the study before 12‐months for non‐PCa‐related reasons (see CONSORT Figure 1A,B) and also did not undergo reassessment. Thus, after excluding these 18 patients from the per protocol population, 76 patients constituted the study cohort for disease re‐assessment at 12‐month (Table 2). Data on PSA, repeat mpMRI, and repeat biopsy were available for 73/76 (96.1%), 47/76 (61.8%) and 60/76 (78.9%), respectively. According to pre‐defined criteria, progression was seen in 7/76 (9.2%), 6/76 (7.9%) and 25/76 (41.7%) according to biochemical (PSA), radiological (mpMRI) or histological (re‐biopsy) criteria, respectively. Overall, at 12 months of the evaluable patients according to any definition, 33/76 (43.3%) had progression. Prolaris® testing was only possible in 21 of 60 (35%) men who underwent re‐biopsy men at 12 month due to a lack of biopsy material.

**TABLE 2:** Patient progression for eligible patients with 12‐month follow up (*n* = 76)

Criteria	No Progression *n* (%)	Progression *n* (%)	Missing *n* (%)	Total *n* (%)
Biochemical (PSA)	66 (86.8)	7 (9.2)	3 (3.9)	76 (100)
Radiological (mpMRI)	41 (53.9)	6 (7.9)	29 (38.2)	76 (100)
Histological (Biopsy)	35 (46.1)	25 (32.9)	16 (21.1)	76 (100)
Any	41 (53.9)	33 (43.3)	2 (2.6)	76 (100)

Abbreviations: mpMRI, multiparametric magnetic resonance imaging; PSA, prostate‐specific antigen.

In total there were six AE and five SAE (Table 3), including one death due to a cardiac event in a patient in the aspirin 300 mg and vitamin D placebo arm. This was deemed to be unrelated to the trial medication by the site Principal Investigator, trial Chief Investigator and the study sponsor. Only two SAE (both rectal bleeding) in one patient taking aspirin 100 mg and active vitamin D were considered drug‐related, and the patient was withdrawn from the study. In total, there were 10 AE/SAEs in the two aspirin arms vs. one in the aspirin placebo arm though only two of these events were deemed related to aspirin. No participant on vitamin D developed hypercalcaemia (defined as serum calcium >2.6 mmol/L), and there was no significant change in serum calcium from baseline to 12 months in either vitamin D or placebo arm (Table S2).

**TABLE 3:** Adverse events (AEs) and serious AE (SAE) in all randomised patients (*n* = 104)

Treatment Arm	AE	SAE	Total Events
Aspirin 300 mg Vitamin D3 (*n* = 17)	0	2 (0)	2 (0)
Aspirin 300 mg Vitamin D3 Placebo (*n* = 16)	2 (0)	1† (0)	3 (0)
Aspirin 100 mg Vitamin D3 (*n* = 19)	1 (0)	2 (2)	3 (2)
Aspirin 100 mg Vitamin D3 Placebo (*n* = 18)	2 (0)	0	2 (0)
Aspirin Placebo Vitamin D3 (*n* = 16)	1 (0)	0	1 (0)
Aspirin Placebo Vitamin D3 Placebo (*n* = 18)	0	0	0 (0)
Total (*n* = 104)	6 (0)	5 (2)	11 (2)

*Note*: Numbers in parentheses represent AEs related to trial intervention.

Abbreviations: AE, adverse event; SAE, serious adverse events.

† Patient died.

## 4 DISCUSSION

Use of minimally‐toxic adjunctive oral drug treatments to reduce disease progression for PCa patients on AS is an important unmet clinical need. Here, we demonstrate feasibility of a multi‐centre randomised, double‐blind, placebo‐controlled trial of aspirin and/or vitamin D3 for newly‐diagnosed PCa patients suitable for AS. We achieve high rates of trial acceptance and entry (80%), tissue biomarker molecular profiling (81%) and treatment compliance (>89%) with low rates of AEs.

Interestingly, we found that overall (i.e., irrespective of treatment allocation), 43.3% showed progression within 12 months of which 9.2%, 7.9% and 41.7% had disease progression according to PSA, mpMRI or biopsy criteria, respectively. This is higher than conversion to RT rates (11.7%) in a recent non‐interventional UK AS cohort,^24^ though others report rates up to 32%.^25^ There are several possible explanations for our findings. Firstly, compared with other non‐interventional AS cohorts, we permitted a higher PSA inclusion threshold (≤15 ng/ml),^15^ leading to a higher baseline PSA (median 7.2 ng/ml),^24^ and observed a higher proportion of ISUP grade group 2 tumours (20.4% vs. 14.5%).^24^ Hence, our study population probably included more aggressive tumours at higher risk of disease progression. Secondly, 44% (46/104) of diagnostic biopsies were performed using a transperineal approach, which increased to 59% (35/60) at the 12‐month reassessment. The transrectal route is known to under‐classify tumours, hence, a change in biopsy technique could have led to a disease re‐classification bias. Thirdly, electing for a composite inclusive disease progression endpoint of any three parameters (biochemical, radiological, and histological), may also lead to the higher progression rates compared to studies using single parameters.^24^

Definitions of disease progression for AS patients can be a challenging aspect of clinical care and research. In day‐to‐day practice, PSA, DRE, mpMRI and re‐biopsy are all routinely used for disease monitoring and to inform RT decisions, but there is limited consensus on choice of progression thresholds.^26^ Though mpMRI has an increasing role in AS,^9^ mpMRI‐based progression without confirmatory biopsy is a new concept, which has not reached an expert consensus.^27^ PSA has a weak link with grade progression, and absolute thresholds for disease progression are controversial.^28^ Histopathological criteria are most commonly used to define progression, of which ISUP grade group progression remains a key marker,^27^ but again thresholds may differ between studies.^26^ Taken together, disparate definitions of disease progression in AS may contribute to variable progression rates observed in published studies.

In our study, we found that aspirin, at both low and high doses, and vitamin D were well‐tolerated separately and in combination, as demonstrated by high treatment compliance in all study arms. No patients developed hypercalcaemia in the vitamin D arm, despite administration at 10 times the daily recommended dose for nutritional support.^29^ We noted only one patient who developed an SAE related to active treatment, in the aspirin arm. These data clearly demonstrate the feasibility, patient acceptability, and safety of delivering minimally‐toxic drugs as adjunctive treatments for men with PCa on AS using aspirin and vitamin D.

Molecular and imaging prognostic biomarkers could help direct adjunctive drug treatments for patients at higher risk of disease progression. For example, mpMRI‐visible disease is associated with a greater likelihood of moving to active treatment at 5 years.^9^ Urinary PCA3 and TMPRSS:ERG expression levels are associated with an increased risk of higher‐grade disease on re‐biopsy for patients on AS.^7^ Using surplus diagnostic and re‐biopsy tissue, we demonstrate the feasibility of Prolaris® CCP testing, which can identify patients at very low disease progression risk^7^ who may not benefit from adjunctive treatments. The Prolaris® test is not expected to be a companion diagnostic for classification of patients to aspirin and/or vitamin D, as the exact molecular basis of action of these agents is unclear. However, this test might serve as a useful prognostic biomarker for PCa patients on AS to assess disease progression risk. The optimal biomarker(s) for risk stratification are yet to be determined, and should be explored via longitudinal outcome‐associated AS studies.

We observed a higher‐than‐anticipated withdrawal rate, with 18 men of out 94 withdrawing before 12 months. Nine of these men withdrew in order to receive RT, despite not meeting PROVENT criteria for disease progression. A further nine patients withdrew for non‐PCa‐related reasons. A limitation of our study is the absence of detailed information captured on the reasons for withdrawals. Patient anxiety is a recognised reason for ceasing AS in favour of RT, which occurs in approximately 10%–36% of cases.^30^ Future studies might consider patient psychological barriers to AS and should aim to include data on eventual treatment decisions as well as final oncological and functional outcomes.

Our feasibility study has clearly shown that adjunctive drug therapies are acceptable, safe and well‐tolerated by patients on AS for PCa. The optimal choice/combination of adjunctive agent is yet to be determined, but should be minimally‐toxic to maximise longer‐term treatment compliance. A UK randomised trial of the 5‐alpha reductase inhibitor finasteride (FINESSE) for low and favourable intermediate risk PCa will soon open to recruitment, with a primary endpoint of cessation of AS based on patient choice, mpMRI, clinical (examination) or biopsy progression, but without biomarker profiling. Future studies should consider selection of patients with higher progression risk and include multiple adjunctive drug agents, and molecular and imaging biomarkers. In addition to data on oncological, functional and quality of life outcomes, qualitative data should also be captured on patient barriers to AS, such as anxiety, reasons for trial withdrawal and drug compliance.

## CONFLICT OF INTEREST

Jack Cuzick receives royalties from and is on the advisory board of Myriad Genetics.

## AUTHOR CONTRIBUTION STATEMENT

The authors listed below have made substantial contributions to the intellectual content of the paper in the sections described below: Conception and design: ED, GS, PC, TO, PR and JC. Acquisition of data: GS, RK, NH, SS, SK, DB, AS and PR. Analysis and interpretation of data: ED, GS, PA, KF, KC, TO, PR and JC. Drafting of manuscript: ED, TO, PR and JC. Critical revision of the manuscript: GS, RK, PA, KF, KC, NH, SS, SK, DM, AS and PC. Statistical Analysis: PA, KF and KC. Obtaining funding: GS, PC, TO, PR and JC. Administrative support, technical or material support: RK, NH. Supervision: GS, PR and JC.

## Supporting information


**Table S1.** Inclusion and Exclusion Criteria.
**Table S2.** Combined change in serum calcium measurement according to Vitamin D arm.
**Table S3.** Treatment compliance as a proportion of dispensed medication that was consumed. Amongst participants who returned their medication packages for independent pharmacist review.
**Table S4.** Patients who returned their medication packages for compliance assessment.
**Table S5**. Reasons for Patient Exclusion (n = 10).
