# Supplementary material for: Feasibility of aspirin and/or vitamin D3 for men with prostate cancer on active surveillance with Prolaris® testing
Source: BJUI Compass. 2022 Jun 11;3(6):458–65. doi: 10.1002/bco2.169 (PMC9579886; doi:10.1002/bco2.169)
Supplement: Supplementary file 1 — Table S1. Inclusion and Exclusion Criteria. Table S2. Combined change in serum calcium measurement according to Vitamin D arm. Table S3. Treatment compliance as a proportion of dispensed medication that was consumed. Amongst participants who returned their medication packages for independent pharmacist review. Table S4. Patients who returned their medication packages for compliance assessment. Table S5. Reasons for Patient Exclusion (n = 10). [file BCO2-3-458-s001.docx]

**PROVENT Supplementary Material 1**

**PROVENT Study Group:**

**Chairman:** Professor Jack Cuzick (Queen Mary University London).

**Urologists:** Mr Sanjeev Madaan (Darent and Gravesham NHS Trust), Miss Jhumur Pati and Mr Abdul M. Chowdhury (Homerton University Hospital NHS Foundation Trust), Mr Brian R.P. Birch and Mr Timothy J. Dudderidge (University Hospitals Southampton NHS Foundation Trust), Professor Caroline M. Moore (University College London Hospitals NHS Foundation Trust and University College London), Mr Alistair D.R. Grey (University College London Hospitals NHS Foundation Trust, University College London, and Barts Health NHS Trust), Mr Kieran P. Jefferson (University Hospitals Coventry and Warwickshire NHS Trust), Professor Howard G. Kynaston (University Hospital of Wales), Mr Gregory L. Shaw and Mr Prabhakar Rajan (Queen Mary University of London, University College London, University College London NHS Foundation Trust, and Barts Health NHS Trust), Professor James S.A. Green (Barts Health NHS Trust), Mr Paul J. Cathcart (Guy’s and St Thomas’ NHS Foundation Trust).

**Histopathologists:** Professor Daniel M. Berney (Queen Mary University of London and Barts Health NHS Trust).

**Oncologists**: Professor Thomas Powles and Professor R. Timothy D. Oliver (Queen Mary University of London and Barts Health NHS Trust).

**Radiologists:** Dr Anju Sahdev (Barts Health NHS Trust)

**Trial co-ordinator**: Roseann Kealy and Victoria Kemp (Queen Mary University of London)

**Trial statisticians**: Panos Alexandris, Kier Finnegan and Kimberly Chu (Queen Mary University London).

**Table S1.** Inclusion and Exclusion Criteria

| **Inclusion Criteria** |
| --- |
| - Male subjects aged 16 years or over with life expectancy > 3 years. - Able to provide written informed consent. - Low or intermediate-risk organ confined prostate cancer (PCa) opting for AS as primary treatment option. - Gleason Grade 3+3 or 3+4 disease on US-guided prostate biopsy. - Organ-confined prostate cancer at the time of diagnosis. - All included men should have had magnetic resonance imaging (MRI) prostate with targeted biopsy of identified lesions as well as systematic biopsy of prostate tissue. - At least ten cores of prostate tissue should be available from the original biopsy procedure to inform histological diagnosis at the time of PCa diagnosis. |
| **Exclusion Criteria** |
| - Previous treatment for PCa (including surgery, radiotherapy, hormone therapy, brachytherapy or focal therapy). - PSA > 15.0ng/ml - Clinical or radiological suspicion of stage T3 or above. - Current enrolment (or within last 30 days) in any other clinical research study. - Current or pre-existing use of either aspirin or vitamin D (> 400 IU/day). - Current or previous use of 5-alpha reductase inhibitors. - Known allergy or hypersensitivity to either NSAIDs of Vitamin D3. - Hypercalcaemia (serum calcium >2.6mmol.L) - Gastrointestinal (GI) contraindications: GI bleeding, peptic ulceration, severe dyspepsia, inflammatory bowel disease, persistent helicobater pylori infection refractory to treatment. - Other medical conditions: haemophilia or bleeding diatheses, chronic kidney disease stage 4 or over, hyperparathyroidism, any active malignancy (i.e. not in remission for fiver years), severe asthma, G6PD deficiency, macular degeneration, tuberculosis, alcohol consumption >4 units regularly for men. - Bowel condition or co-existing medical condition that would make repeat trans-rectal or trans-perineal prostate biopsy hazardous or difficult to perform (e.g. prior bowel surgery, recto-urethral fistula, conditions requiring continuous anti-coagulation). - Concomitant use of any of the medications interacting with aspirin or vitamin D. |

**Table S2.** Combined change in serum calcium measurement according to Vitamin D arm.

| **Treatment arm** | **n** | **Baseline**  **mmol/l, median (IQR)** | **n** | **Change in median at 12-months, nmol/L (IQR)** |
| --- | --- | --- | --- | --- |
| *Active/ Vitamin D* | 52 | 2.32 (2.27, 2.38) | 32 | +0.02 (-0.04, 0.08) |
| *Placebo* | 51 | 2.34 (2.28, 2.43) | 37 | -0.01 (-0.06, 0.04) |
| **Total** | 103 |  | 69 |  |

**Table S3.**  Treatment compliance as a proportion of dispensed medication that was consumed amongst participants who returned their medication packages for independent pharmacist review.

| **Follow up visit** | **Month 0-6 (%)**  **n=64** | **Month 6-12 (%) n=49** |
| --- | --- | --- |
| *Aspirin - Tablets Taken* | | |
| Placebo | 90 | 91 |
| 100mg | 92 | 91 |
| 300mg | 93 | 91 |
| All | 91.7 | 91 |
| *Vitamin D - Oil bottles consumed* | | |
| Placebo | 80 | 88 |
| Active | 98 | 94 |
| All | 89 | 91 |

**Table S4.** Patients who returned their medication packages for compliance assessment.

| **Follow up visit** | **Months 0-6* (%)** | **Months 6-12** (%)** |
| --- | --- | --- |
| *Aspirin* | | |
| Placebo | 26/32 (81.2) | 17/27 (63) |
| 100mg | 18/34 (52.9) | 11/22 (50) |
| 300mg | 20/28 (71.4) | 19/25 (76) |
| **Total** | 64/94 (68.1) | 47/76 (61.8) |
| *Vitamin D* | | |
| Placebo | 30/46 (63.2) | 24/40 (60) |
| Active | 34/48 (70.8) | 25/36 (69.4) |
| **Total** | 64/94 (68.1) | 49/76 (64.5) |

*Not including participant exclusions. **Not including participant exclusions nor withdrawals.

**Table S5**. Reasons for Patient Exclusion (n=10).

| **Number of patients** | **Reason** |
| --- | --- |
| 1 | Death during study. |
| 2 | Pre-entry MRI out of date |
| 1 | Gleason Grade Group 3. |
| 3 | PCa diagnosis ‘out of date’ meaning the investigations providing diagnosis of PCa and eligibility >12 months from enrolment into trial. |
| 2 | Development of condition incompatible with study. |
| 1 | 1 never started |
